# Gut Microbiota Dysbiosis Induced by Decreasing Endogenous Melatonin Mediates the Pathogenesis of Alzheimer’s Disease and Obesity

**DOI:** 10.3389/fimmu.2022.900132

**Published:** 2022-05-10

**Authors:** Boqi Zhang, Tong Chen, Maosheng Cao, Chenfeng Yuan, Russel J. Reiter, Zijiao Zhao, Yun Zhao, Lu Chen, Wenjing Fan, Xin Wang, Xu Zhou, Chunjin Li

**Affiliations:** ^1^ College of Animal Sciences, Jilin University, Changchun, China; ^2^ Department of Cellular and Structural Biology, University of Texas Health Science Center, San Antonio, TX, United States

**Keywords:** alzheimer's disease, microbiota dysbiosis, obesity, gut- brain axis, melatonin, fecal microbiota transplantation, systemic inflammation

## Abstract

Lifestyle choices, external environment, aging, and other factors influence the synthesis of melatonin. Although the physiological functions of melatonin have been widely studied in relation to specific organs, the systemic effects of endogenous melatonin reduction has not been reported. This study evaluates the systemic changes and possible pathogenic risks in an endogenous melatonin reduction (EMR) mouse model deficient in the rate limiting enzyme in melatonin production, arylalkylamine N-acetyltransferase *(Aanat)* gene. Using this model, we identified a new relationship between melatonin, Alzheimer’s disease (AD), and gut microbiota. Systematic changes were evaluated using multi-omics analysis. Fecal microbiota transplantation (FMT) was performed to examine the role of gut microbiota in the pathogenic risks of EMR. EMR mice exhibited a pan-metabolic disorder, with significant transcriptome changes in 11 organs, serum metabolome alterations as well as microbiota dysbiosis. Microbiota dysbiosis was accompanied by increased gut permeability along with gut and systemic inflammation. Correlation analysis revealed that systemic inflammation may be related to the increase of *Ruminiclostridium_5* relative abundance. 8-month-old EMR mice had AD-like phenotypes, including Iba-1 activation, A β protein deposition and decreased spatial memory ability. Moreover, EMR mice showed decreased anti stress ability, under high-fat diet, EMR mice had greater body weight and more obvious hepatic steatosis compared with WT group. FMT improved gut permeability, systemic inflammation, and AD-related phenotypes, while reducing obesity in EMR mice. Our findings suggest EMR causes systemic changes mediated by gut microbiota dysbiosis, which may be a pathogenic factor for AD and obesity, we further proved the gut microbiota is a potential target for the prevention and treatment of AD and obesity.

## Introduction

Melatonin—originally defined as a neurohormone secreted by the pineal gland (1), is synthesized in the retina, platelets, skin, lymphocytes, uterus, and importantly, the gut, in which the amount of melatonin is approximately 400 times greater than that in the pineal gland (2). The melatonin synthesis pathway consists of consecutive enzymatic steps. Arylalkylamine N-acetyltransferase (AANAT), a key rate-limiting enzyme in the pathway, catalyzes the conversion of serotonin to N-acetyl serotonin, which is subsequently converted to melatonin by acetylserotonin O-methyltransferase (3). Clinical and experimental results show that melatonin—which has antioxidant, anti-inflammatory, and anticancer properties—mitigates the effects on metabolic syndrome, slows development of induced neurodegenerative diseases, and reduces obesity (4). Melatonin is a potential therapeutic agent for Alzheimer’s disease (AD); moreover, it stimulates neural plasticity and alleviates some neuropathologies of AD by regulating autophagy and sleep (5, 6). Also, the level of endogenous melatonin negatively correlates with cognitive deficits. Lifestyle choices, external environment, aging, and other factors affect the synthesis of melatonin (7–9). However, the specific relationship between endogenous melatonin level and neurodegenerative diseases remains to be determined.

The gut microbiota influences various physiological processes, including the immune response and autophagy. Dysregulation of the gut microbiota causes several pathological abnormalities, including organ development (10). Microbiota dysbiosis is closely associated with gut barrier function; with a increase in gut permeability, the movement of pathogenic bacteria and harmful metabolites from the intestine to the systemic circulation, contributing to the pathogenesis of many diseases, especially inflammation-related diseases (11). For example, microbiota dysbiosis increases gut permeability and aggravates the severity of mastitis caused by *Staphylococcus aureus* in mice, which is reversed by fecal microbiota transplantation (FMT) (12); similar findings have been reported in the context of acute pancreatitis (13) and endometritis (12). Importantly, both neuroinflammation and systemic inflammation have been confirmed in the pathogenesis of AD (14), with the inflammatory process in AD involving gut microbiota (15). Thus, clinical, and experimental results suggest the gut microbiota as an attractive therapeutic target for a series of diseases. However, to the best of our knowledge, the relationship between melatonin, gut microbiota, and AD has not been adequately examined.

Herein, using a melatonin-deficient mouse model we evaluated the systemic changes that may relate to gut microbiota dysbiosis the pathogenesis of AD and obesity. For this study, we utilized transcriptomic, metabolomic, and phenotypic analyses to clarify the pathophysiological link among melatonin, gut microbiota, and the systemic diseases of interest.

## Materials and Methods

Detailed descriptions of the protocols for the glucose and insulin tolerance tests, immunofluorescence assay, RNA-sequencing, 16S rDNA sequencing, *in vivo* gut permeability analysis, T-maze test, and FMT methods are available in the **Supplementary Material** section online.

### Animals and Ethics Approval

All animal experiments were approved by the Animal Ethics Association of Jilin University (JLU : SY201909022). All experimental animals were purchased from Cyagen Biosciences (Suzhou, China). The endogenous melatonin reduction (EMR) mouse model was established using 10-week-old male C57BL/6N Aanat-knockout mice (*Aanat^−/−^*) (**Figure S1**). All animals were raised in the specific pathogen-free animal lab of Jilin University (25 ± 2°C; relative humidity, 45–60%; light:dark cycle, 12:12 h) with free access to food and drinking water. Considering the influence of environmental factors on gut microbiota, all mice were strictly retained in Specific pathogen Free (SPF) level laboratory; mice used for fecal collection and FMT test were each fed in separate cages.

Samples for RNA-seq, 16S rDNA-seq, and serum metabolomics were obtained from 10-week-old mice. AD-related phenotypic analysis and FMT were performed using 8-month-old adult male C57BL/6N mice (32.5 ± 3.6 g). 10-week-old adult male C57BL/6N mice were used for the establishment of the obese mouse model (high-fat and normal diet composition is shown in **Tables S1**, **S2**) and subsequent phenotypic analyses.

### Tissue Immunohistochemistry

Tissue samples were obtained from 10-week-old adult mice (24.43 ± 2.7 g). The mice were anesthetized using tribromomethane before surgically removing the brain, heart, liver, kidney, lung, skin, stomach, spleen, testis, skeletal muscle, and epididymal white adipose tissue (EWAT; n = 3-5). Tissues were fixed overnight with 4% paraformaldehyde, washed with methanol, ethanol, ethanol/xylene (1:1), and xylene, and then embedded in paraffin. The paraffin block was cut into 5-µm-thick slices and stained with hematoxylin-eosin using standard methods (16). For liver Oil Red O staining, frozen sections were thawed to room temperature (23°C ± 2°C), washed with 4% paraformaldehyde for 1 min and with 60% isopropanol for 3 min, and dipped in Oil Red O solution for 15 min. The sections were stained with hematoxylin for 20 s after washing with distilled water and sealed with glycerol gelatin after moderate washing for image acquisition and analysis.

### Reverse Transcription-Quantitative Polymerase Chain Reaction

TRIzol (Takara, Japan) was used to extract total RNA from the brain, skin, liver, lung, and testis according to the manufacturer’s instructions. cDNA synthesis was performed using PrimeScript RT Reagent Kit (Takara Bio, Japan). SYBR green-based qPCR assays (segment 1: 95°C for 10 min; segment 2: 95°C for 30 s, 60°C for 1 min, 55 cycles; segment 3: 95°C for 1 min, 55°C for 30 s, 95°C for 30 s) were performed using an Agilent Mx3005p (Agilent, Foster City, CA, USA) thermocycler (20 µL assay volume) in a two-step reaction program. The relative expression level of mRNA was calculated by the 2^-ΔΔCt^ method (17), with glyceraldehyde 3-phosphate dehydrogenase (*Gapdh*) as the internal reference gene. The primer sequences are provided in **Table S3**.

### Metabolomics Analysis

Samples were analyzed using ultra-high performance liquid chromatography-mass spectrometry as reported previously (18). The acquired mass spectral data (peak picking, peak grouping, retention time correction, second peak grouping, and annotation of isotopes and adducts) were obtained using XCMS software (Sciex 3.4.1). Raw data files were converted into mzXML format and processed by the XCMS, CAMERA, and MetaX toolbox using R software. Each ion was identified by combining the retention time (RT) and *m/z* data. A three-dimensional matrix containing arbitrarily assigned peak indices (RT‐*m/z* pairs), sample names (observations), and ion intensity information (variables) was generated.

Metabolites were annotated by matching the exact molecular mass (*m/z*) of samples to the Kyoto Encyclopedia of Genes and Genomes (KEGG) and HMDB databases. A match was only considered if the mass difference between the observed and the database values was <10 ppm. The molecular formula of the metabolite was validated by the isotopic distribution measurements and an in-house fragment spectrum library of metabolites. The intensity of peak data was processed by MetaX; features that were detected in <50% of quality control (QC) samples or <80% of biological samples were removed. The remaining peaks with missing values were imputed with the k‐nearest neighbor algorithm to improve data quality. Principal coordinate analysis (PCoA) was performed for outlier detection and batch effects evaluation using the pre‐processed dataset. QC-based robust locally estimated scatterplot smoothing signal correction was fitted to the QC data with respect to the order of injection to minimize signal intensity drift over time. QC samples with a relative standard deviation of the metabolic features >30% were removed.

### Serum Biochemical Measurements

Mouse blood samples were centrifuged at 4,500 × *g* for 10 min to obtain the serum, which was diluted 1:4 with the dilution solution. The diluted solution (50 μL) was tested using a Bio-Plex Pro Mouse Cytokine Grp I Panel 23-plex kit (Bio-Rad, Austin, TX, USA) at Wayne Biotechnology (Shanghai) Co., Ltd. according to a previous report (19) with a bead suspension platform (Bio-Plex MAGPIX System, Bio-Rad). Considering the circadian rhythm of melatonin secretion, we collected serum samples at 4 am (GMT+8). Mouse melatonin levels were measured using enzyme-linked immunosorbent assay kits (ELISA; Yuanxin Biotechnology Co., Ltd., Shanghai, China), and T-tau, interleukin (IL)-6, MCP-1, tumor necrosis factor (TNF)-α, and LCN2 levels were also measured using ELISA kits (Longdeng Biotechnology Co., Ltd., Shanghai, China). The uric acid serum level was tested using the uric acid assay kit (Nanjing Jiancheng Biological Engineering Research Institute, China).

### Statistical Analysis

Relative abundance of gut microbiota, serum marker levels, and fat weight were compared between groups using the unpaired student’s *t*-test (SPSS 23 software, IBM, Armonk, NY, USA). Data are expressed as the mean ± SEM and *P* < 0.05 was considered statistically significant. Comparisons among more than two groups were made with one-way analysis of variance, followed by Dunnett’s test.

Student’s two-tailed *t*‐tests were conducted to detect differences in metabolite levels between phenotypes; the *P* value was adjusted for multiple tests using the false discovery rate (FDR) according to the Benjamini-Hochberg method. Supervised partial least squares-discriminant analysis was conducted through MetaX to discriminate the different variables between groups using the variable importance in the projection (VIP) value. A VIP cut‐off value of 1.0 was used to select important features.

For linear discriminant analysis (LDA) effect size (LEFse), the Kruskal Wallis rank sum test was used to detect significantly different species, and the Wilcoxon rank sum test was used to detect whether all subspecies of the significantly different species converged to the same classification level. LDA was used to obtain the final differential species (i.e., biomarker). A clustering correlation heatmap with signs was drawn using the OmicStudio tools (https://www.omicstudio.cn) and Pearson correlation analysis (R version 3.6.1).

## Results

### Reduced Endogenous Melatonin Levels Caused Metabolic Disorder

**Figure 1:**
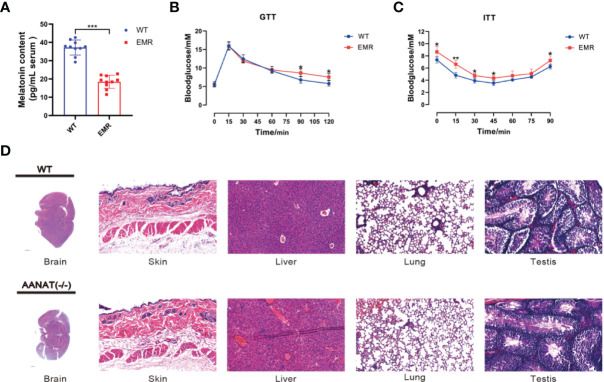
Reduced endogenous melatonin levels caused metabolic disorders in EMR mice. **(A)** Serum melatonin level in *Aanat^−/−^* and wild-type (WT) mice (n =10). **(B)** Blood glucose level using the glucose tolerance test (GTT) (n = 7). **(C)** Blood glucose level using the insulin tolerance test (ITT) (n = 7). **(D)** Hematoxylin and eosin staining of the mouse brain (1000 μm), skin (50 μm), liver (50 μm), lungs (50 μm), and testis (50 μm) (n = 3-5). Differences between EMR and WT mice were assessed by Student’s *t-*test: *P < 0.05, **P < 0.01, ***P < 0.001.

The serum melatonin level in *Aanat^−/−^* mice was significantly lower than that in wild type (WT) mice (*P* < 0.001, **Figure 1A**), confirming that the EMR mouse model was successfully established. The blood glucose level in EMR mice was significantly higher than that in WT mice at 90 and 120 min (*P* < 0.05, **Figure 1B**), and EMR mice exhibited insulin tolerance (**Figure 1C**). However, by histological observation, no pathological features were observed in the brain, heart, liver, kidney, lung, skin, stomach, spleen, testis, skeletal muscle, and EWAT of EMR mice (**Figures 1D**, **S2A**). Similarly, no significant differences in the body weight or litter number were found between EMR and WT mice (*P* > 0.05, **Figure S2B**).

### EMR Altered Gene Expression in 11 Organs

**Figure 2:**
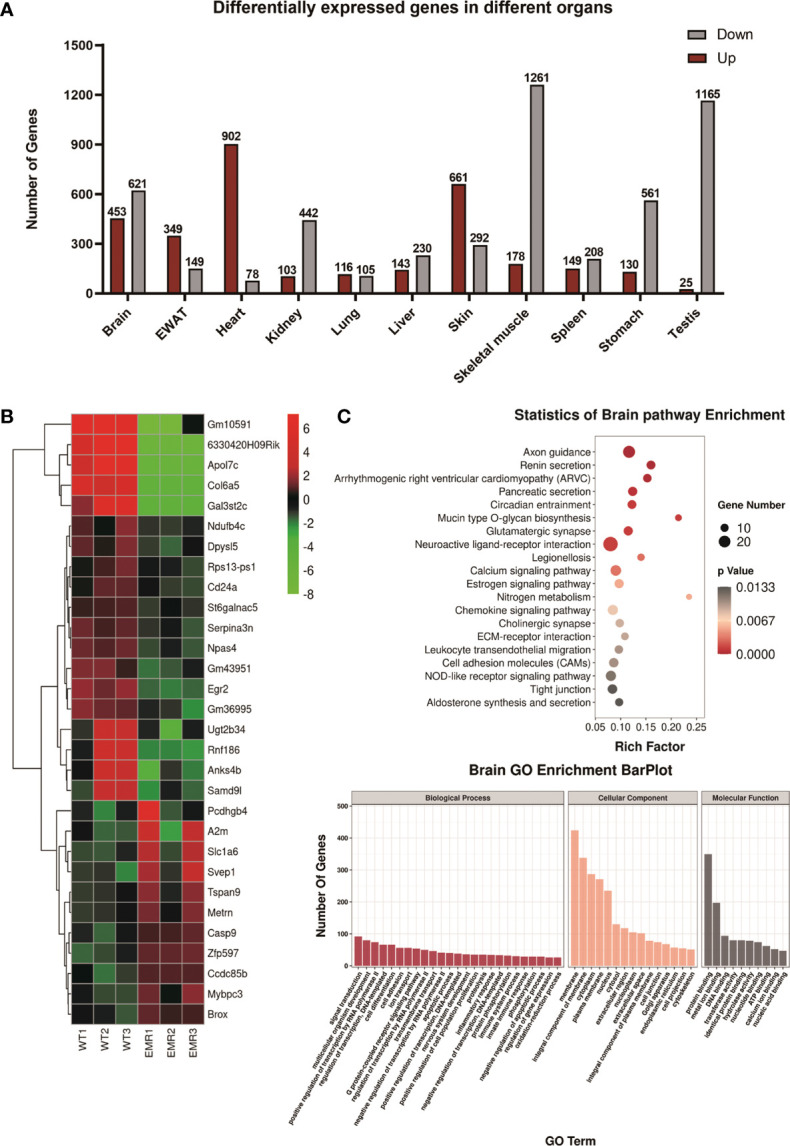
Transcriptome analysis of EMR and wild-type (WT) mice. **(A)** Overall differentially expressed genes (DEGs) in 11 organs (fold change >2 and *P* < 0.05). **(B)** Top 30 (The lowest *p*-values) up- or down-regulated DEGs in the brain represented by a heatmap. **(C)** Gene Ontology (GO) enrichment bar plot of DEGs in the brain. Kyoto Encyclopedia of Genes and Genomes (KEGG) pathway enrichment analysis of DEGs with the lowest *P*-values.

In total, 8,321 differentially expressed genes (DEGs) were found in 11 organs, with 3,209 and 5,112 genes up- and down-regulated in EMR mice, respectively (**Figures 2A, B**, **S3A**). The enriched Gene Ontology (GO) terms and top 20 (the lowest *p* value) KEGG pathways of the DEGs in each organ are shown in (**Figures 2C** and **S3B, C**).

In the brain, KEGG enrichment analysis showed that axon guidance, calcium signaling pathway, circadian entrainment, and cholinergic synaptic physiology were significantly enriched; these pathway functions were related to nervous system diseases, especially degenerative dysfunction, such as AD and Parkinson’s disease (20–23). In the skin, the pathways were significantly enriched in melanogenesis, phosphatidylinositol 3‐kinase protein kinase B (PI3K-AKT), and nuclear factor kappa B (NF-κB) signaling pathways (**Figure S3C**), all of which are associated with melanoma (24–26). In the liver, pathways were mostly enriched in metabolic pathways (**Figure S3C**) along with those associated with non-alcoholic liver disease (NAFLD), including retinol metabolism, peroxisome proliferator-activated receptor (PPAR), and forkhead box O (FoxO) signaling pathways (27) (**Figure S3C**). Further analysis found that after EMR, the variation of the relative expression of some DEGs were consistent with its variation in the pathogenesis and poor prognosis of tissue-specific diseases, including NAFLD, melanoma, and lung cancer (**Figure S3D**). Some of the disease-related DEGs identified in brain, skin, liver, testis, and lung (**Figure S3D**) were further verified *via* qPCR (**Figure S4**). Overall, the results suggest that EMR may have potential links with a variety of diseases, although further investigations are warranted.

### EMR Altered Metabolites and Metabolic Pathways

**Figure 3:**
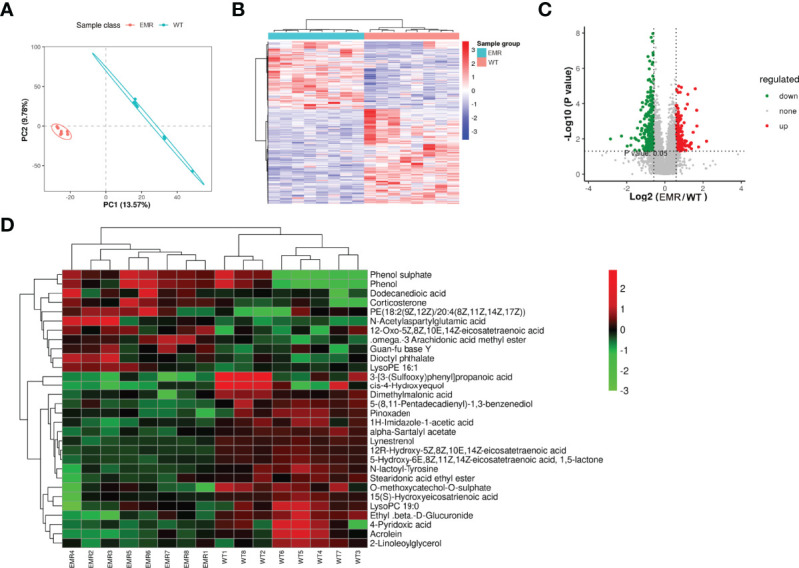
EMR changed the serum metabolic pathways and serum metabolites. **(A)** PLS-DA scores plot. **(B)** Metabolic ions heatmap with red and blue blocks indicating an increase and decrease in relative abundance, respectively. **(C)** Volcano plot showing increased (red) and decreased (green) levels of serum metabolic ions with gray circles indicating no significant changes. **(D)** Heatmap of changed metabolites in two groups.

The PLS-DA analysis uncovered differences between the two groups (**Figure 3A**). In total, 497 metabolic ions (**Figures 3B, C**) and 30 metabolites (**Figure 3D**) were detected (MS2Metabolite). Pathway topology analysis identified 16 significantly-enriched metabolic pathways (*P* < 0.05, **Table 1**).

**Table 1:** Significantly enriched metabolic pathways in endogenous melatonin reduction mice.

Pathway	P value	FDR	Pathway ID
Glycerophospholipid metabolism	4.03095512473011e-06	3.6278596122571e-05	map00564
Prion diseases	1.55265217942604e-05	6.98693480741719e-05	map05020
Glycosylphosphatidylinositol (GPI)-anchor biosynthesis	1.55265217942604e-05	6.98693480741719e-05	map00563
Autophagy - animal	3.10141491584041e-05	0.000111650936970255	map04140
Choline metabolism in cancer	0.000281812676967333	0.000845438030901998	map05231
Regulation of lipolysis in adipocytes	0.00053531451525391	0.00137652303922434	map04923
Retrograde endocannabinoid signaling	0.000867435794328781	0.00195173053723976	map04723
Aldosterone synthesis and secretion	0.00139131886037758	0.00278263772075515	map04925
Alanine, aspartate and glutamate metabolism	0.00189596390440326	0.00310248638902352	map00250
Vitamin B6 metabolism	0.00189596390440326	0.00310248638902352	map00750
Protein digestion and absorption	0.00529428423108775	0.00794142634663162	map04974
Arachidonic acid metabolism	0.0131210295125879	0.0181675793251217	map00590
Tyrosine metabolism	0.0141456374821687	0.0181872481913597	map00350
Drug metabolism - cytochrome P450	0.01742371028089	0.020908452337068	map00982
Steroid hormone biosynthesis	0.0222550950276348	0.0250369819060892	map00140
Neuroactive ligand-receptor interaction	0.0369961151328568	0.0391723571994954	map04080
Metabolic pathways	0.380611697294188	0.380611697294188	map01100

Idms2 Serum differential metabolites.

### EMR Induced to Gut Microbiota Dysbiosis and Increased Inflammatory Marker Levels

**Figure 4:**
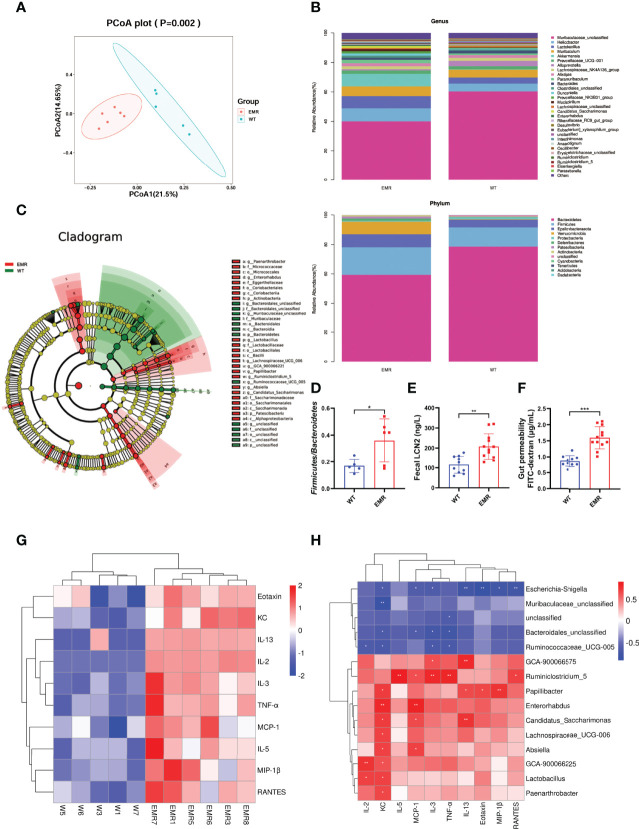
EMR led to gut microbiota dysbiosis and systemic inflammation. **(A)** PCoA plot. **(B)** Gut microbiota relative abundance and composition at the phylum and genus levels (n = 5–6). **(C)** Cladogram using LEfSe to identify biomarkers to distinguish between the groups. **(D)** *Firmicutes/Bacteroidetes* ratio. **(E)** Fecal LCN2 level (n = 10-12). **(F)** Serum FITC-dextran level to measure gut permeability (n = 10-12). **(G)** Heat map of significantly changed cytokines in EMR (*P* < 0.05): IL-2, IL-3, IL-5, IL-13, KC, MCP-1, RANTES, TNF-α, eotaxin, and MIP-1β (n = 5–6). **(H)** Pearson correlation heatmap between genera with significant relative abundance changes and cytokines. *P < 0.05, **P < 0.01, ***P < 0.001.

The richness and number of species in the gut microbiome were not significantly different between EMR and WT mice based on the Simpson, Shannon, Chao1, and observed indices (**Figure S5A**). However, PCoA plots detected overall differences in beta-diversity (microbial composition and structure) between the groups (**Figure 4A**). The total phylum, class, order, family, and genus compositions of microbiota in EMR mice were different than those in WT mice (**Figures 4B**, **S5B**). The LEFse identified distinct gut microbiota in EMR and WT mice with biomarkers at all levels (**Figure 4C**). The relative abundance of *Bacteroidetes* significantly decreased in EMR mice (*P* < 0.05, **Figure S5C**). Moreover, EMR mice had a significantly increased relative abundance of *Lactobacillus* (*P* < 0.05, **Figure S5C**), and there was a significant difference in the relative abundance of *Firmicutes/Bacteroidetes* between the groups (*P* < 0.05, **Figure 4D**).

The level of the fecal gut inflammation marker LCN2 increased significantly (*P* < 0.01) in the EMR mice (**Figure 4E**), and the FITC test confirmed that the gut permeability increased significantly compared with that of WT group (*P* < 0.001, **Figure 4F**). In general, EMR mice showed gut microbiota dysbiosis, gut inflammation, and increased gut permeability.

As gut microbiota dysbiosis and leaky gut are important factors inducing chronic systemic inflammation, we measured serum cytokine levels (13). Among 23 cytokines tested, the levels of ten significantly differed between the groups (*P* < 0.05); the levels of the pro-inflammatory cytokines TNF-α and MCP-1 significantly increased (*P* < 0.05), indicating that EMR promotes systemic inflammation in mice (**Figure 4G**). We investigated the correlation between genera with significant (*P* < 0.05) differences in relative abundance and cytokines (**Figure 4H**). Pearson correlation analysis showed that *Ruminiclostridium_5* (**Figure S5D**) was positively correlated with TNF-α (*P* < 0.01) and MCP-1 (*P* < 0.05). These results indicate that the change in pro-inflammatory factors may be related to the relative abundance of *Ruminiclostridium_5.*

### EMR Caused AD-Related Phenotypes

**Figure 5:**
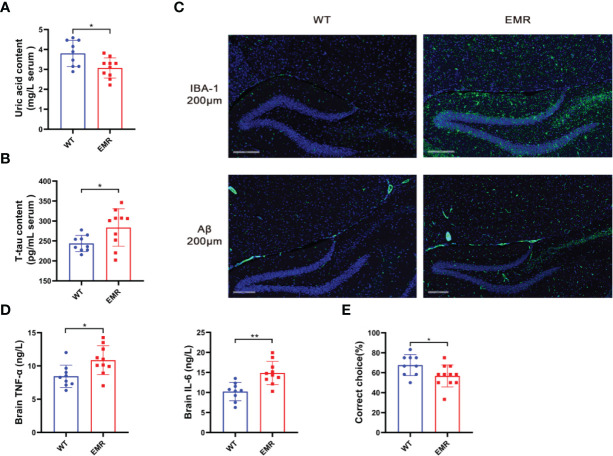
EMR led to an Alzheimer’s disease-like phenotype in 8-month-old mice. **(A)** Serum uric acid level (n = 9–10). **(B)** Serum t-tau level (n = 9–10). **(C)** Changes in IBA-1 and Aβ positive area in hippocampal slices from two groups. Representative fluorescent images are presented. The scale bar represents 200 µm (n = 2-5). **(D)** Brain TNF-α and IL-6 levels (n = 9–10). **(E)** Correct rate in the mouse T-maze test (n = 9–11). Differences were assessed using Student’s *t-*test: *P < 0.05, **P < 0.01.

The relative abundance of *Lactobacillus* was negatively correlated with the level of serum uric acid (28). As patients with AD typically have low uric acid levels and a pathogenic risk of systemic inflammation contributing to AD (29), we verified the AD phenotype of 8-month-old EMR mice. The serum uric acid level in EMR mice was significantly lower than that in WT mice at the same age (*P* < 0.05, **Figure 5A**), and EMR mice had significantly higher serum levels of T-tau, a marker of prodromal states in mild AD (30) (**Figure 5B**). Microglia activation and Aβ protein deposition were observed in the brains of EMR mice, which were not detected in WT mouse brains (**Figure 5C**). Moreover, the levels of the inflammatory factors IL-6 and TNF-α in the brains of EMR mice were significantly higher than those in WT mice (*P* < 0.05, **Figure 5D**). T-maze was used to test the spatial memory ability of animals; the results showed that the spatial learning ability of EMR mice was decreased (*P* < 0.05, **Figure 5E**). Thus, 8-month-old EMR mice showed an AD-like phenotype, suggesting that a decrease in endogenous melatonin levels contributes to the pathogenesis of AD.

### EMR Aggravated High-Fat Diet-Induced Obesity

**Figure 6:**
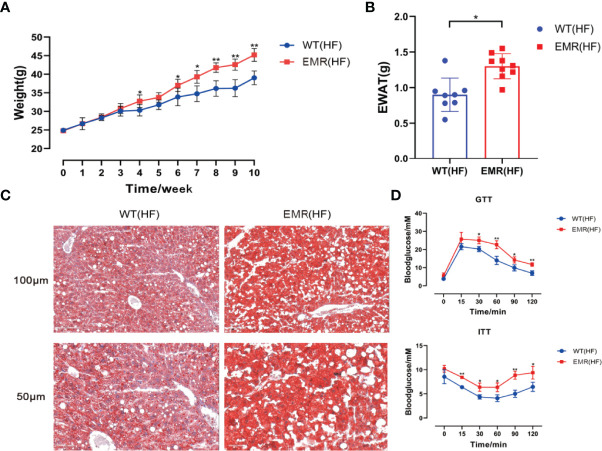
EMR will aggravate the degree of obesity induced by high-fat diet. **(A)** Changes in body weight of mice in the two groups measured every week, high-fat feeding for 10 weeks (n = 8–10). **(B)** Epididymal white adipose tissue (EWAT) weight (n = 8–9). **(C)** Oil Red O staining showing that a marked increase in hepatic steatosis in EMR group. The scale bar represents 100 µm and 50 µm (n = 3-5). **(D)** Blood glucose level measured using the GTT and ITT (n = 7–9). Differences were assessed using Student’s *t-*test: *P < 0.05,**P < 0.01

Metabolic disorders and gut microbiota dysbiosis are closely related to the pathogenesis of obesity. Therefore, in addition to systemic changes, we studied the resistance of EMR mice to external insults. The 10-week-old mice in the EMR and WT groups were fed a high-fat diet for 10 weeks. From the fourth week, the weight of EMR mice was significantly higher than that of WT mice until the end of the tenth week (*P* < 0.01, **Figure 6A**). The EWAT weight of EMR mice was significantly higher than that of WT mice (*P* < 0.05, **Figure 6B**). Oil Red O staining showed a marked increase in hepatic steatosis in the EMR group (**Figure 6C**). EMR mice showed more severe insulin resistance (*P* < 0.05) and glucose tolerance (*P* < 0.01) than WT mice (**Figure 6D**). Overall, EMR mice showed a more obesity-prone phenotype, suggesting that the decrease in endogenous melatonin levels contributes to obesity.

### FMT Improved Gut Permeability, Systemic Inflammation, AD-Like Phenotype, and Obesity in EMR Mice

**Figure 7:**
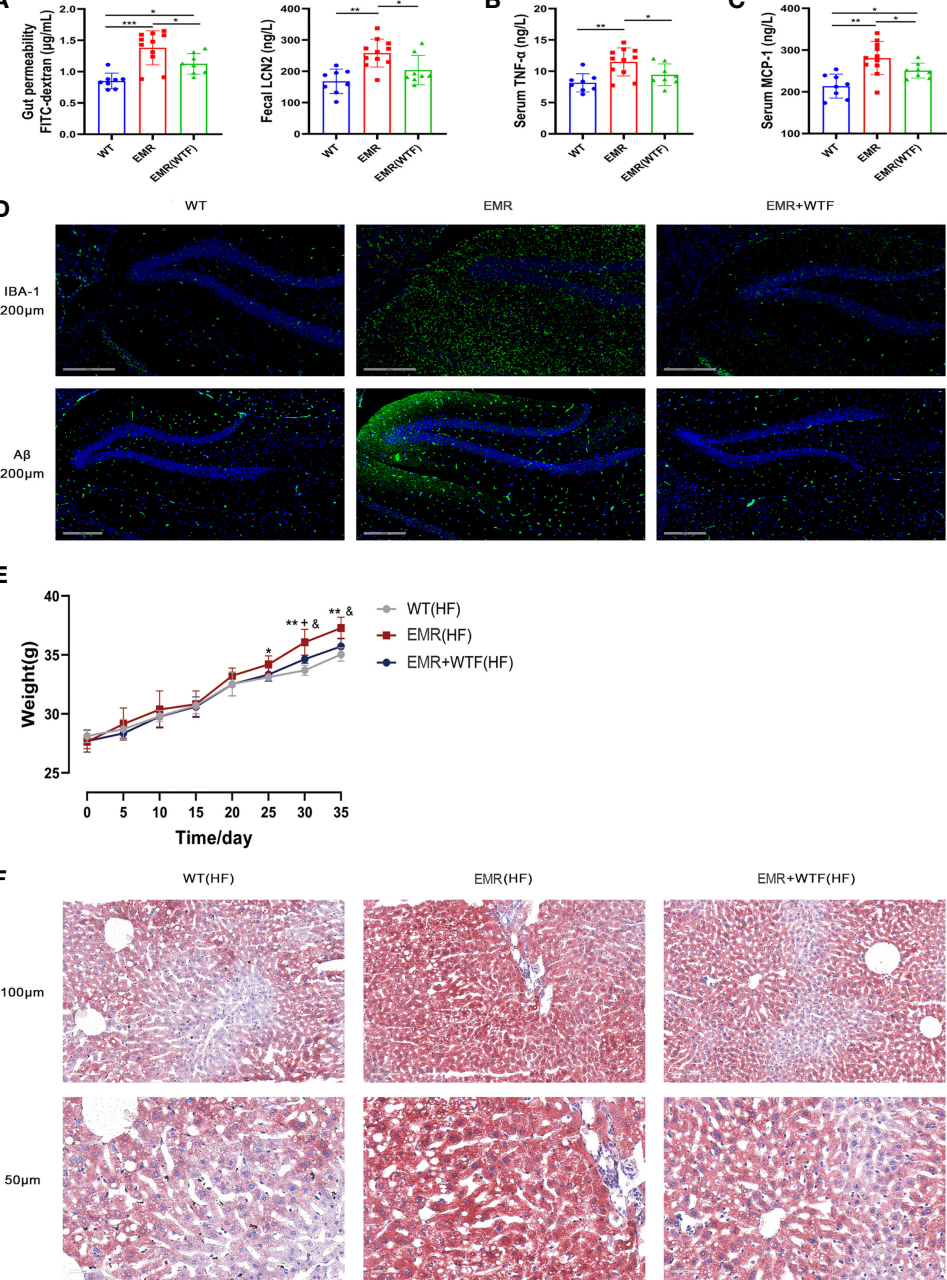
Fecal microbiota transplantation (FMT) can improve gut permeability, systemic inflammation, Alzheimer’s disease (AD)-like phenotype, and obesity in EMR mice. **(A)** Serum FITC-dextran (n = 8–11) and fecal LCN2 (n = 8–11) levels. **(B)** Serum TNF-α level (n = 8-11). **(C)** Serum MCP-1 level (n = 8-11). **(D)** Changes in IBA-1 and Aβ positive area in hippocampal slices from three groups. Representative fluorescent images are presented. The scale bar represents 200 µm (n = 3). **(E)** Changes in body weight of the mice in the two groups measured every 5 days, high-fat feeding for 35 days (n = 7–10). **(F)** Oil Red O staining showing a marked attenuation in HFD-induced hepatic steatosis in EMR+WTF mice. The scale bar represents 100 µm and 50 µm (n = 3). For more than two-group comparisons, one-way ANOVA followed by Dunnett’s test was performed. *P < 0.05, **P < 0.01, WT vs EMR; ^&^ *P* < 0.05, EMR vs EMR+WTF; + *P* < 0.05, WT vs EMR+WTF.

FMT from WT mice significantly improved the gut permeability of 8-month-old EMR mice, significantly reduced the level of fecal LCN2 (*P* < 0.05, **Figure 7A**), and significantly lowered the levels of serum pro-inflammatory factors TNF-α and MCP-1 (*P* < 0.05, **Figures 7B, C**). FMT also reversed the microglia activation and Aβ protein deposition in the brains of EMR mice (**Figure 7D**).

FMT significantly reduced the body weight (*P* < 0.05, **Figure 7E**), and alleviated the glucose tolerance test (GTT) and insulin tolerance test (ITT) values (**Figure S6**) of EMR mice fed a high-fat diet. Oil Red O staining showed a marked attenuation in hepatic steatosis (**Figure 7F**). In summary, the gut microbiota mediates the effects of EMR on gut permeability, systemic inflammation, the AD-like phenotype, and the obesity-prone phenotype.

## Discussion

Herein, we first established an animal model of decreased melatonin synthesis and EMR in *Aanat^−/−^* mice to explore the systemic changes in EMR from a variety of omics angles. The results demonstrate that EMR-mediated gut microbiota dysbiosis contributes to the pathogenesis of AD and obesity.

Changes in the composition of gut microbiota as a result of the EMR state have not been described despite evidence that melatonin supplementation can affect the composition of gut microbiota. Following EMR, the gut microbiota composition of mice changed significantly, with a reduced relative abundance of *Bacteroidetes* and a change in the *Firmicutes/Bacteroidetes* ratio. Contrary to a previous report showing a positive correlation between melatonin and *Lactobacillus* (31), we observed an increase in the relative abundance of *Lactobacillus* in EMR mice. Moreover, we observed a significant increase in gut permeability (leaky gut) and systemic inflammation in EMR mice. Gut microbiota dysbiosis, leaky gut, and bacterial translocation are important factors inducing systemic inflammation; a long-term leaky gut triggers the immune response and leads to a chronic inflammatory state (32).

**Figure 8:**
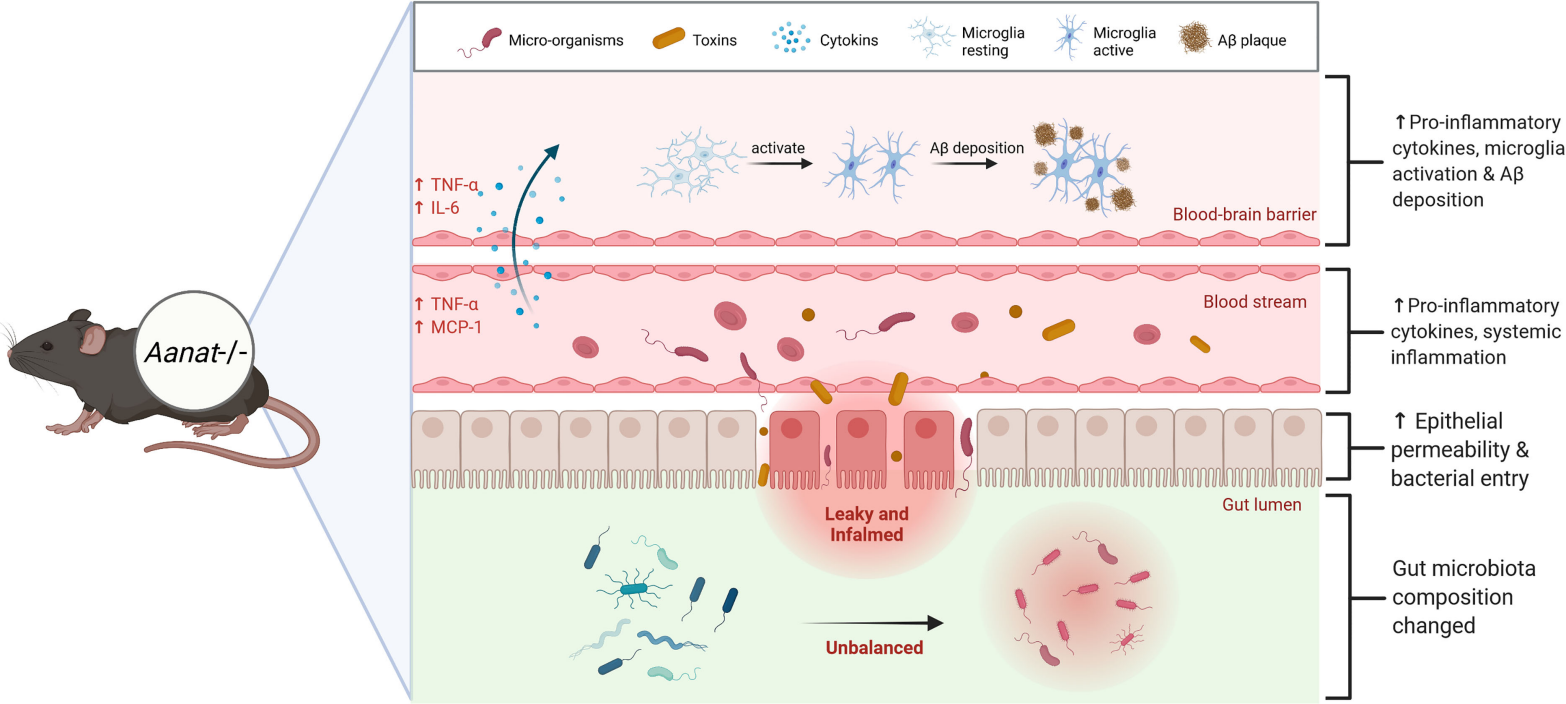
Endogenous melatonin reduction causes the gut microbiota dysbiosis, accompanied by gut inflammation and increased gut permeability. Because of the leaky gut, micro-organisms and toxins in the gut enter the blood circulation, increasing blood pro-inflammatory factors TNF-α and MCP-1, resulting in chronic systemic inflammation. A state of chronic systemic inflammation alters the tight junctions and damages the cells that constitute the blood-brain barrier, facilitating the active transfer of pro-inflammatory factors from the blood into the brain. Eventually leads to Alzheimer’s disease (AD)-like phenotype, microglia activation and Aβ protein deposition.

Gut microbiota dysbiosis-mediated systemic inflammation is closely related to AD pathogenesis (13). A state of chronic systemic inflammation alters the tight junctions and damages the cells that constitute the blood-brain barrier (33), facilitating the active transfer of pro-inflammatory factors from the serum into the brain (34). TNF-α and IL-6 levels were significantly up-regulated and microglia were activated in the brain of EMR mice. Microglia activation and Aβ deposition are important pathological features of AD (35) identified in the brains of EMR mice. Microglia are activated through soluble Aβ oligomers, fibers, or lipopolysaccharide binding, which are then transformed into a pro-inflammatory M1 phenotype, leading to the up-regulated expression of APP, BACE1, and γ-secretase complex to ultimately cause the deposition of Aβ (**Figure 8**) (36). Importantly, we conducted a correlation analysis on the significantly different genera and cytokines in the gut and serum of 2-month-old mice; *Ruminiclostridium_5* was positively correlated with inflammatory markers TNF-α and MCP-1. An over-abundance of *Ruminiclostridium* is positively correlated with the activation of paranuclear ventricular microglia (37). Thus, *Ruminiclostridium* may play an important role in the gut–brain interaction induced by EMR; however, the specific mechanism requires further study.

The EMR also exhibited a significant reduction in serum uric acid level and significant rise in T-tau protein level, a marker of prodromal state in mild AD (30). Uric acid has antioxidant properties and neuroprotective effects; AD is usually accompanied by low uric acid levels (38). The increased relative abundance of *Lactobacillus* is negatively correlated with the level of serum uric acid (39). Thus, we speculate that the low uric acid level of EMR mice may be mediated by *Lactobacillus*. However, since we did not perform further detect the relative abundance of *Lactobacillus* in 8-month-old mice, this hypothesis requires further study. Nevertheless, FMT significantly decreased serum TNF-α and MCP-1 levels, alleviating microglia activation and protein deposition, suggesting that EMR-induced systemic inflammation and the AD-like phenotype are mediated by gut microbiota.

The therapeutic effect of melatonin on obesity and its complications have been extensively studied (39); however, the aggravation of obesity caused by EMR has not been previously reported. FMT reversed the exacerbation of obesity in EMR mice and reduced hepatic steatosis, indicating that the exacerbation of obesity in EMR mice was also mediated by the gut microbiota.

Gut microbiota participates in the host digestion of proteins and carbohydrates, and changes in the composition of the microbiota can regulate the expression of genes related to lipid absorption (40). Short-chain fatty acids, important metabolites of gut microbiota, are essential in regulating energy homeostasis (41). Butyrate and acetate have been established as agents that alleviate lipid deposition and steatosis (42). Importantly, melatonin regulates lipid metabolism in mice fed a high-fat diet by shaping the gut microbiota and increasing the acetic acid level (43). However, the specific mechanism of EMR in exacerbating obesity needs further experimental research.

By means of transcriptome and metabolome analyses, we found that after EMR, genes and metabolites related to the pathogenesis and poor prognosis of NAFLD, melanoma, male infertility and lung cancer changed. Metabolic disorders are typical features of NAFLD. KEGG enrichment analysis of the liver showed that retinol metabolism, the PPAR signaling pathway, and the FoxO signaling pathway were significantly enriched in EMR mice (**Figure S3**). These pathways have been shown to directly drive increases in free fatty acids and contribute to fatty liver associated diseases (27). The expression of cytochrome P450 omega-hydroxylase 4A14 (CYP4A14), which catalyzes the hydroxylation of medium-chain fatty acids and arachidonic acid in mice, is significantly up-regulated in EMR mice (**Figure S4**). It is highly expressed in both patients with NAFLD and mice as it augments the accumulation of lipids in the hepatocytes (44). Serum corticosterone levels are also increased in high-fat diet-induced NAFLD rats and human patients (45), consistent with the present findings (**Table 1**). Melatonin exerts its anticancer activity on hepatocellular carcinoma (HCC) cells through its anti-proliferation and pro-apoptotic effects (46). The expression level of suppressor of cytokine signaling 3 (SOCS3) in HCC cells decreases, and deletion of SOCS3 accelerates hepatocyte proliferation and promotes hepatitis-induced hepatocarcinogenesis (47). Serum amyloid 1 (SAA1) expression is down-regulated in liver tumors and associated with poorer survival of patients with HCC (48). Changes of gene expression after EMR were consistent with those in the disease (**Figure S4**).

Melanoma is a highly malignant tumor derived from melanocytes, which differentiate *via* melanogenesis regulated by melanocortin 1 receptor (MC1R), tyrosinase (TYR), TYR-related protein-1 (TYRP1), and dopachrome tautomerase (DCT). Melatonin reduced melanogenesis in human melanoma cells *in vitro* (49). The expression of regulatory factor genes was significantly up-regulated in skin after EMR (**Figure S4**). The overexpression or inhibition of MC1R expression enhanced or reduced melanoma migration in mice, respectively (50). TYRP1 expression was negatively correlated with survival time in patients with metastatic melanoma (51). In addition to the melanogenesis pathway, the PI3K-AKT and NF-κB pathways play essential roles in the pathogenesis and drug resistance of melanoma (25, 26).

In addition, the regulatory changes of keratin (*Krt*) 17, 18 (52), *Wnt5a* (53), matrix metallopeptidase (*Mmp*) 13 (54), and *Malt1* (55) after EMR were consistent with their changes during the pathogenesis and poor prognosis of melanoma (**Figure S4**). Melatonin inhibits the metastasis of lung cancer and potentially synergizes with drugs in the treatment of non-small-cell lung cancer. The lung transcriptomic data of EMR mice show changes in the expression of genes previously identified in lung cancer cells, including metastasis associated in lung denocarcinoma transcript 1 (*Malat1*) (56), synaptotagmin 7 (*Syt7*) (57), and tubulin beta 3 class III (*Tubb3*) (58) (**Figure S4**).

Melatonin has a protective action on male reproductive function. In addition to the regulatory changes in spermatogenesis-related genes (**Figure S3**), the downregulation of R-spondin 1 (*Rspo1*), piakophilin 2 (*Pkp2*), and Eps15-homology domain-containing protein (*Ehd4*) were observed in EMR mice (**Figure S4**). The number of Sertoli cells decreased and testicular dysplasia as observed in *Wnt4* ^−/−^ male mice; *RSOP1* is a regulator of early gonadal cell proliferation in both sexes (59). Knockdown of connexin 43 (*Cx43*) and *Pkp2* expression in male rats results in the transient perturbation of the Sertoli cell tight junction barrier (60). *Ehd4^−/−^* male mice exhibit a 50% reduction in testicular weight, increased germ cell apoptosis, and reduced fertility (61). Although this evidence suggests that EMR mice may have reproductive abnormalities, male EMR mice were not infertile.

In conclusion, we determined the systemic changes after EMR and clarified the possible relationship between EMR and disease. Our study suggests that EMR mediates AD and aggravates obesity through gut microbiota, indicating that melatonin not only assists in maintaining physiological functions but also improves stress resistance. The findings also suggest EMR as a pathogenic factor contributing to the physiological deterioration associated with AD and obesity. Importantly, this work provides insights into gut microbiota as a new therapeutic target of diseases and provides evidence for the role of melatonin in human diseases.

## Data Availability Statement

The data presented in the study are deposited in the NCBI repository, accession number GSE194203 and PRJNA 799188.

## Ethics Statement

The animal study was reviewed and approved by Animal Ethics Association of Jilin University (JLU : SY201909022).

## Author Contributions

XZ, CL, BZ contributed to the conception of the study. RR perform the analysis with constructive discussions. TC, ZZ, YZ, LC performed the experiment. MC, CY, WF, XW contributed to analysis and manuscript preparation. All authors contributed to the article and approved the submitted version.

## Funding

This work was supported by the National Natural Science Foundation of China (32172726 and 31301969) and the Key Research and Development Program of Jilin Province (20210202103NC and 20210202048NC).

## Conflict of Interest

The authors declare that the research was conducted in the absence of any commercial or financial relationships that could be construed as a potential conflict of interest.

## Publisher’s Note

All claims expressed in this article are solely those of the authors and do not necessarily represent those of their affiliated organizations, or those of the publisher, the editors and the reviewers. Any product that may be evaluated in this article, or claim that may be made by its manufacturer, is not guaranteed or endorsed by the publisher.
