# Supplementary material for: Gut Microbiota Dysbiosis Induced by Decreasing Endogenous Melatonin Mediates the Pathogenesis of Alzheimer’s Disease and Obesity
Source: Front Immunol. 2022 May 10;13:900132. doi: 10.3389/fimmu.2022.900132 (PMC9127079; doi:10.3389/fimmu.2022.900132)
Supplement: Supplementary file 1 [file DataSheet_1.docx]

Supplementary Material

# Supplementary Methods

**1.1 Glucose and insulin tolerance tests**

The glucose tolerance test (GTT) and insulin tolerance test (ITT) were performed using a Roche ACCU-CHEK blood glucose meter after mice were fasted for 12 and 4 h, respectively. The mice received intraperitoneal injection of 2 g glucose or 1 U insulin (dissolved in saline)/kg body weight and measurements were recorded at 0, 15, 30, 60, 90, and 120 min.

**1.2 Immunofluorescence assay**

The tissue slices were fully washed with phosphate-buffered saline (PBS) and patted to remove excess water; a water blocking ring was drawn with a PAP pen to prevent the sample from drying, and the wet box was sealed at room temperature for 10 min after dropping peroxidase sealing solution. The sections were incubated with the following primary antibodies at room temperature followed by incubation at 4 ℃ overnight: mouse anti-Aβ42 (1:300; cat# 805501, Biolegend) and rabbit anti-AIF1 (9A3) (1:300; bsm-54132R Bioss). After washing in PBS, the corresponding rabbit and mouse secondary antibodies were added dropwise, and DAPI staining and sealing were performed after microwave treatment. Sections were scanned on a Leica aperio versa system (Leica Microsystems) for image analysis.

**1.3 RNA-sequencing**

TRIzol (Takara, Japan) was used to extract total RNA according to the manufacturer’s instructions. After library construction, 2 × 150 bp paired-end sequencing was performed using the Novaseq 6000 system (Illumina, San Diego, CA, USA) following the manufacturer protocol. Cutadapt software (version 1.9) was used to remove low-quality reads, adapter contamination, and undetermined bases. HISAT2 software (version 2.0.4) was used to map reads to the genome (<ftp://ftp.ensembl.org/pub/release-101/fasta/mus_musculus/dna/>) and the reads were assembled using StringTie (version 1.34d). The results from all samples were merged using Gffcompare software (version 0.98) to reconstruct a comprehensive transcriptome. The expression levels of all transcripts were calculated in FPKM units [total exon fragments/mapped reads (millions) × exon length (kB)] using StringTie and Ballgown. Differentially expressed mRNAs with fold change > 2 or < 0.5 and a P value < 0.05 were selected by the R package edgeR (enriched), and pathway enrichment analysis was performed using the Gene Ontology (GO; http://geneontology.org) and Kyoto Encyclopedia of Genes and Genomes (KEGG; http://www.kegg.jp/kegg) databases.

**1.4 16S rDNA sequencing**

Fecal samples were frozen in liquid nitrogen and stored at –80 °C. DNA was extracted using the E.Z.N.A. stool DNA kit (Omega Bio-Tek, USA). The 5′ ends of the primers were tagged with specific barcodes per sample and sequenced using universal primers 341F (5’-CCTACGGGNGGCWGCAG-3’), 805R (5’-GACTACHVGGGTATCTAATCC-3’) . PCR amplification (25 ng template in a 25 µL reaction volume) of prokaryotic 16S fragments consisted of an initial denaturation step at 98 °C for 30 s; 32 cycles of denaturation at 98 °C for 10 s, annealing at 54 °C for 30 s, and extension at 72 °C for 45 s; and a final extension at 72 °C for 10 min. The PCR products were purified using AMPure XP beads (Beckman Coulter Genomics, Danvers, MA, USA) and quantified with a Qubit fluorometer (Invitrogen, USA). The amplicon pools were prepared for sequencing, and the size and quantity of the amplicon library were assessed on a 2100 Bioanalyzer Instrument (Agilent, Santa Clara, CA, USA) and the KAPA library quantification kit for Illumina (Kapa Biosystems, Woburn, MA, USA), respectively.

Samples were sequenced as described in the preceding section. Paired-end reads were assigned to samples based on their unique barcode and truncated by cutting off the barcode and primer sequence. Paired-end reads were merged using the Fast Length Adjustment of SHort reads (FLASH) program. Quality filtering of the raw reads was performed using fqtrim (version 0.94) with fic filtering conditions to obtain high-quality clean tags. Chimeric sequences were filtered using Vsearch software (version 2.3.4) and then dereplicated using DADA2 to corrects Illumina-sequenced amplicon errors. It mainly removes the background noise of abiotic sequences, repeated sequence information, and the sequences whose filter length is lower than the threshold (400bp). Alpha and beta diversities were analyzed using QIIME2 and normalized to the same sequences randomly. Alpha diversity was assessed with the Chao1, observed species, Goods coverage, Shannon, and Simpson indices. Sequence alignment was performed using BLAST and the sequences were annotated with the SILVA (release 132) database. All diagrams were generated using R packages (version 3.5.2).

**1.5 Gut permeability analysis *in vivo***

The gut permeability of 10-week-old mice was measured using the fluorescein isothiocyanate-dextran (FITC-D) method. The mice were fasted for 6 h and then gavaged with 500 mg/kg FITC-D (4000 MW, Sigma-Aldrich) diluted with normal saline. After 4 h, the blood was collected from the orbital venous plexus, and centrifuged at 4 ℃ and 12,000 rpm for 10 min to extract the serum. The fluorescence intensity was analyzed by a fluorescence spectrophotometer (excitation wavelength: 485 nm, emission wavelength: 535 nm) and the FITC-D standard curve was drawn to calculate the concentration.

**1.6 T-maze test**

T-maze was used to test the spatial memory ability of animals. The test was conducted at 8:00-12:00. All animals were acclimatized to the testing room for 1 h before the test. A T-maze with two short arms (25 × 8 cm) and a long arm (30 × 8 cm, 7 cm high) was constructed. The mice were initially placed in the starting arm and allowed to arbitrarily select the left and right target arms; all four claws had to enter the arm to determine successful selection. If a mouse chose to lower the baffle, it could stay in the target arm for 30 s before being placed back to the starting arm for 5 s, and then the above process was repeated. The test was repeated 12 times for each mouse to calculate the alternation percentage.

**1.7 Fecal microbiota transplantation (FMT)**

Fecal samples collected from 8-month-old wild-type (WT) mice were transplanted to 8-month-old EMR mice (EMR+WTF); 10-week-old WT mice fecal microbiota were transplanted to 10-week-old high-fat diet EMR mice (EMR+WTF(HF)). All fecal samples were frozen at –80 ℃. After thawing, 100 mg of the fecal sample from different ages WT mice (WTF) was accurately weighed, diluted with 2 mL normal saline (0.05 g/mL), fully mixed, centrifuged at 800 × *g* for 3 min, and the supernatant was collected and used as transplant material. Each EMR mouse was gavaged with the transplant material at 100 μL once a day for 2 weeks, and then regularly maintained for another 3 weeks.

# Supplementary Figures and Tables

**2.1 Supplementary Tables**

**Table S1.** High-fat (60FDC) purified rodent diet.

| **Ingredient** | **kcal/g** | **gram** | **Kcal** | **gm%** | **kcal%** |
| --- | --- | --- | --- | --- | --- |
| Caisein | 4 | 200 | 800 | – | – |
| L-Cystine | 4 | 3 | 12 | – | – |
| Sucrose | 4 | 68.8 | 275 | – | – |
| Dyetrose | 4 | 125 | 500 | – | – |
| Lard | 9 | 245 | 2,205 | – | – |
| Soybean Oil | 9 | 25 | 225 | – | – |
| Cellulose | 0 | 50 | 0 | – | – |
| Mineral Mix # 210088 | 1.6 | 10 | 16 | – | – |
| Calcium Carbonate | 0 | 5.5 | 0 | – | – |
| Dicalcium Phosphate | 0 | 13 | 0 | – | – |
| Potassium Citrate H_2_O | 0 | 16.5 | 0 | – | – |
| Vitamin Mix # 300050 | 3.9 | 10 | 39 | – | – |
| Choline Bitartrate | 0 | 2 | 0 | – | – |
| Blue Dye | 0 | 0.05 | 0 | – | – |
| Protein | – | – | – | 26 | 20 |
| Carbohydrate | – | – | – | 26 | 20 |
| Fat | – | – | – | 35 | 60 |
| Kcal/gm | – | – | – | 5.26 | |

**Table S2.** Nutritional components of basic normal feed for mice.

| **Vitamin** |  | **Mineral** |  | **Amino acid** |  |
| --- | --- | --- | --- | --- | --- |
| Vitamin A  (IU/kg) | 7800.00 | Sodium  (g/kg) | 3.10 | Methionine + cystine(g/kg) | 5.80 |
| Vitamin D  (IU/kg) | 1200.00 | Magnesium  (g/kg) | 2.90 | Lysine  (g/kg) | 8.90 |
| Vitamin E  (mg/kg) | 67.00 | Potassium  (g/kg) | 7.40 | Tryptophan  (g/kg) | 2.10 |
| Vitamin K  (mg/kg) | 5.00 | Copper  (mg/kg) | 11.40 | Arginine  (g/kg) | 9.90 |
| Vitamin B1  (mg/kg) | 10.00 | Iron  (mg/kg) | 113.70 | Leucine  (g/kg) | 14.80 |
| Vitamin B2  (mg/kg) | 15.00 | Manganese  (mg/kg) | 80.00 | Isoleucine  (g/kg) | 7.40 |
| Vitamin B6  (mg/kg) | 10.00 | Zinc  (mg/kg) | 31.60 | Threonine  (g/kg) | 6.60 |
| Vitamin B12  (mg/kg) | 0.02 | Selenium  (mg/kg) | 0.20 | Valine  (g/kg) | 8.90 |
| Niacin  (mg/kg) | 55.00 | Iodine  (mg/kg) | 0.70 | Histidine  (g/kg) | 4.90 |
| Pantothenic acid  (mg/kg) | 22.00 | _ | _ | Phenylamine + tyrosine(g/kg) | 14.60 |
| Biotin  (mg/kg) | 0.20 | _ | _ | _ | _ |
| Choline  (mg/kg) | 1250.00 | _ | _ | _ | _ |
| Folic acid  (mg/kg) | 6.60 | _ | _ | _ | _ |
| **Energy ratio** | | | | | |
| Protein | 23.07% | | | | |
| Fat | 11.85% | | | | |
| Carbohydrate | 65.08% | | | | |
| Total energy | 3.40kcal/g | | | | |

**Table S3.** Oligonucleotide primers used in quantitative polymerase chain reaction analysis.

| **Gene** | **Primer sequences** |
| --- | --- |
| *Gapdh* | Forward: 5ʹ-GTGTTTCCTCGTCCCGTAG-3ʹ |
|  | Reverse: 5ʹ-ACAATCTCCACTTTGCCACT-3ʹ |
| *Cyp4a14* | Forward: 5ʹ -TGCAGAAGGCCAGGAAGAAG-3ʹ |
|  | Reverse: 5ʹ-GCACCTCCTCTCTGCATCTC-3ʹ |
| *Saa1* | Forward: 5ʹ-GCAGGATGAAGCTACTCACCA-3ʹ |
|  | Reverse: 5ʹ-CATAGTTCCCCCGAGCATGG-3ʹ |
| *Socs3* | Forward: 5ʹ -TGTCGGAAGACTGTCAACGG-3ʹ |
|  | Reverse: 5ʹ-CCGTTGGGGCTGGATTTTTG-3ʹ |
| *Mt1* | Forward: 5ʹ-TCCTGTCTGTGTACCGCAAC-3ʹ |
|  | Reverse: 5ʹ-CGAGGTCTGCCACAGCTAAA-3ʹ |
| *Mt2* | Forward: 5ʹ-GAAGGGCTCTTTGTCACCAGTTAC-3ʹ |
|  | Reverse: 5ʹ-GGTTCAGGAGCCCATAAACAAT-3ʹ |
| *Mc1r* | Forward: 5ʹ-CAAGCAGCGGCAGAAAAAGT-3ʹ |
|  | Reverse: 5ʹ-CTGGCCAAGGTTACGGATGT-3ʹ |
| *Tyrp1* | Forward: 5ʹ-CGGTCTTTGACGAATGGCTAA-3ʹ |
|  | Reverse: 5ʹ-CGTTTTCCAACGGGAAGGTA-3ʹ |
| *Tyr* | Forward: 5ʹ-ACACACTGGAAGTATTTTTGAACA-3ʹ |
|  | Reverse: 5ʹ-TAGGTGCATTGGCTTCTGGG-3ʹ |
| *Dct* | Forward: 5ʹ-TCAGAGCTCGGGCTCAGTTT-3ʹ |
|  | Reverse: 5ʹ-TGTTCAGCACGCCATCCA-3ʹ |
| *Krt17* | Forward: 5ʹ-TAGTGGCCTGGGTAGTGCTC-3ʹ |
|  | Reverse: 5ʹ-CACCTTGTCCAGGTAGGAGGC-3ʹ |
| *krt18* | Forward: 5ʹ-TGCCAGCTCTGGATTGACTG-3ʹ |
|  | Reverse: 5ʹ-GTTCCTCGCGGTTCTTCTGA-3ʹ |
| *Wnt5a* | Forward: 5ʹ-ATTTCCACGCTATACCAACTCCT-3ʹ |
|  | Reverse: 5ʹ-ATTCCTTGATGCCTGTCTTCG-3ʹ |
| *mmp13* | Forward: 5ʹ-GACCCCAACCCTAAGCATCC-3ʹ |
|  | Reverse: 5ʹ-CCTCGGAGACTGGTAATGGC-3ʹ |
| *Malt1* | Forward: 5ʹ-GGACAAAGTCGCCCTTTTGAT-3ʹ |
|  | Reverse: 5ʹ-TCCACAGCGTTACACATCTCA-3ʹ |
| *Hoxc13* | Forward: 5ʹ-GGAAGTCTCCCTTCCCAGAC-3ʹ |
|  | Reverse: 5ʹ-CTGGCTGCGTACTCCTTCTC-3ʹ |
| *Malat1* | Forward: 5ʹ-CTGACCCAGGTGCTACACAG-3ʹ |
|  | Reverse: 5ʹ-CGCTTGCTCCTCAGTCCTAG-3ʹ |
| *Syt7* | Forward: 5ʹ-CGAAGGGGACCATGTACCG-3ʹ |
|  | Reverse: 5ʹ-CCGCAGAGGACGATAGTGAC-3ʹ |
| *Tubb3* | Forward: 5ʹ-CCCTTCGATTCCCTGGTCA-3ʹ |
|  | Reverse: 5ʹ-ACGGCACCATGTTCACAGC-3ʹ |
| *Nr4a1* | Forward: 5ʹ-TGTGAGGGCTGCAAGGGCTTC-3ʹ |
|  | Reverse: 5ʹ-AAGCGGCAGAACTGGCAGCGG-3ʹ |
| *Rspo1* | Forward: 5ʹ-GAGGCGGAAATGCAAGATCG-3ʹ |
|  | Reverse: 5ʹ-ACTCCATGGTGCTGTTAGCG-3ʹ |
| *Pkp2* | Forward: 5ʹ-ACGAAGATGTTCAACGGGCT-3ʹ |
|  | Reverse: 5ʹ-CCGAGGCACTCCATTCAGTT-3ʹ |
| *Ehd4* | Forward: 5ʹ-TACCGCTTCCACGAGTTTCAC-3ʹ |
|  | Reverse: 5ʹ-GAATCTGTGGTTGGCTCTGGA-3ʹ |

## Supplementary Figures


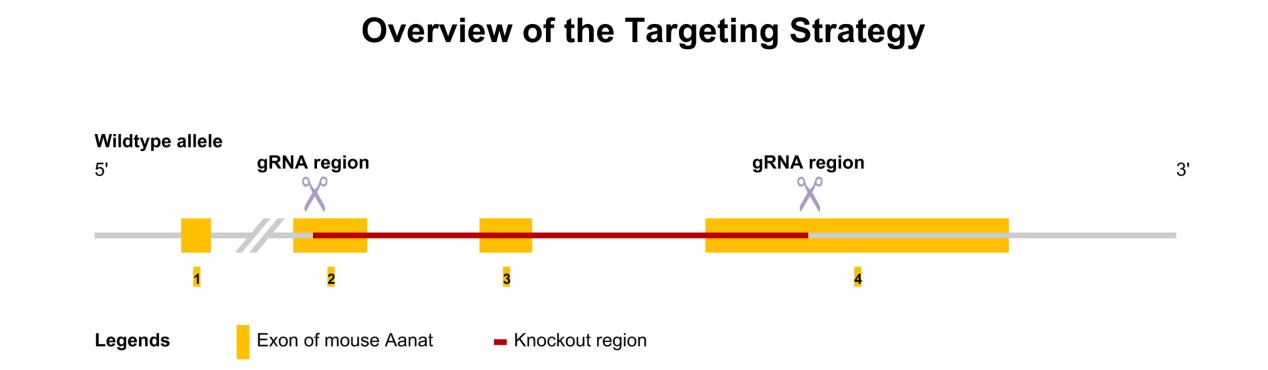


**Figure S1. Overview of the targeting strategy.** *Aanat* knockout (*Aanat^-/-^*) mice (EMR mice) generated by CRISP/Cas9 and purchased from Cyagen. The strategy for generating the mutant mice is schematically shown. The *Aanat* gene (NCBI reference sequence: NM_009591; Ensembl: ENSMUSG00000020804) is located on mouse chromosome 11.4. The ATG start codon is in exon 2 and the TGA stop codon is in exon 4 (transcript: ENSMUST00000153476). Exons 2–4 were selected as the target site, which covers 100.0% of the coding region. The knockout region does not include any other known gene. Cas9 and gRNA are co-injected into fertilized eggs for EMR mouse production.

**
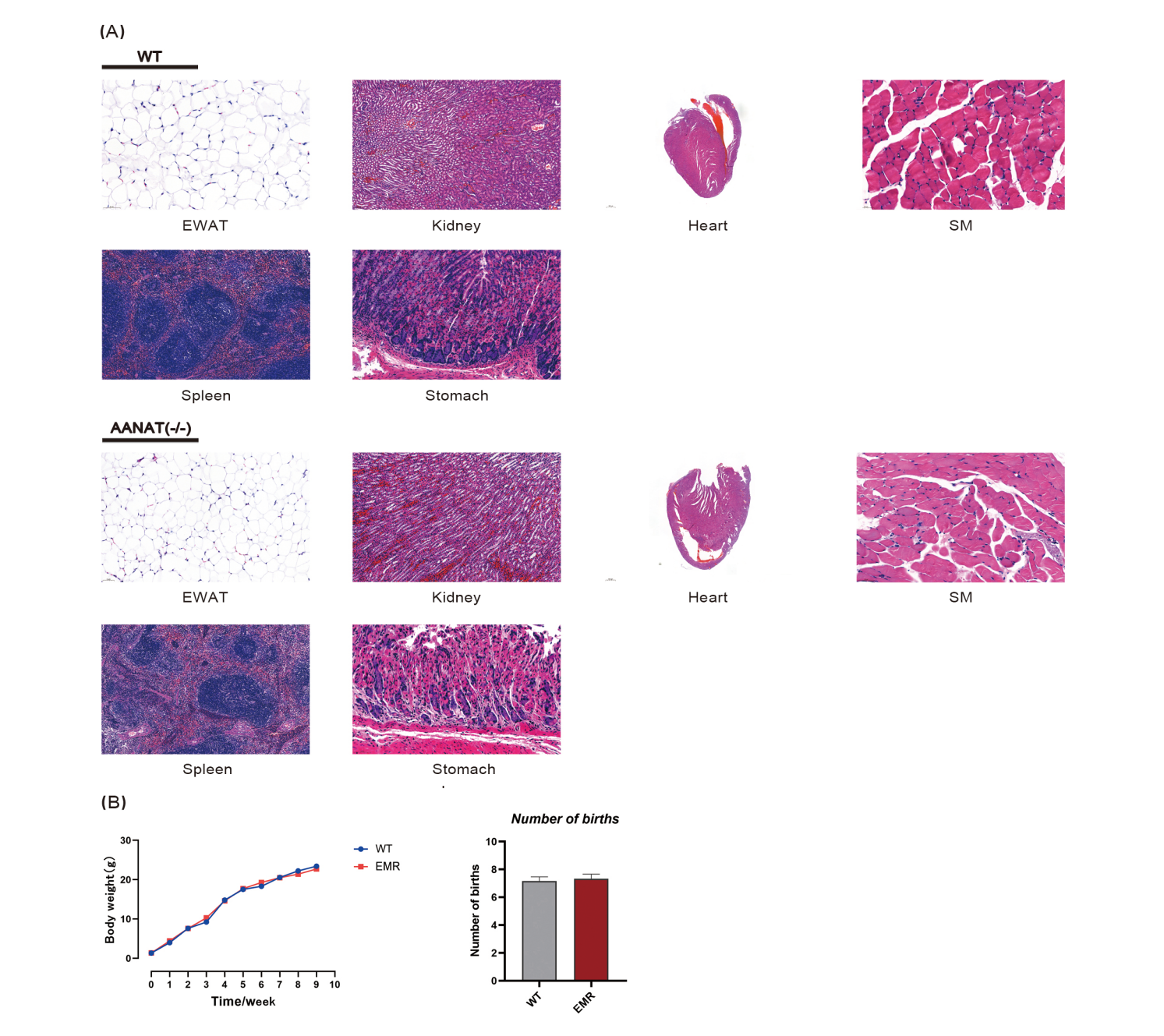
**

**Figure S2.** Pathological sectioning of mouse organs. (A) Hematoxylin-eosin (HE) staining of the epididymal white adipose tissue (EWAT; 50 μm), kidney (50 μm), heart (1,000 μm), skeletal muscle (50 μm), spleen (50 μm), and stomach (50 μm) (n = 3). (B) Body weight (n = 8) and birth rate (n = 5) of 2-month-old mice; mice were caged at a 1 male:2 female ratio.


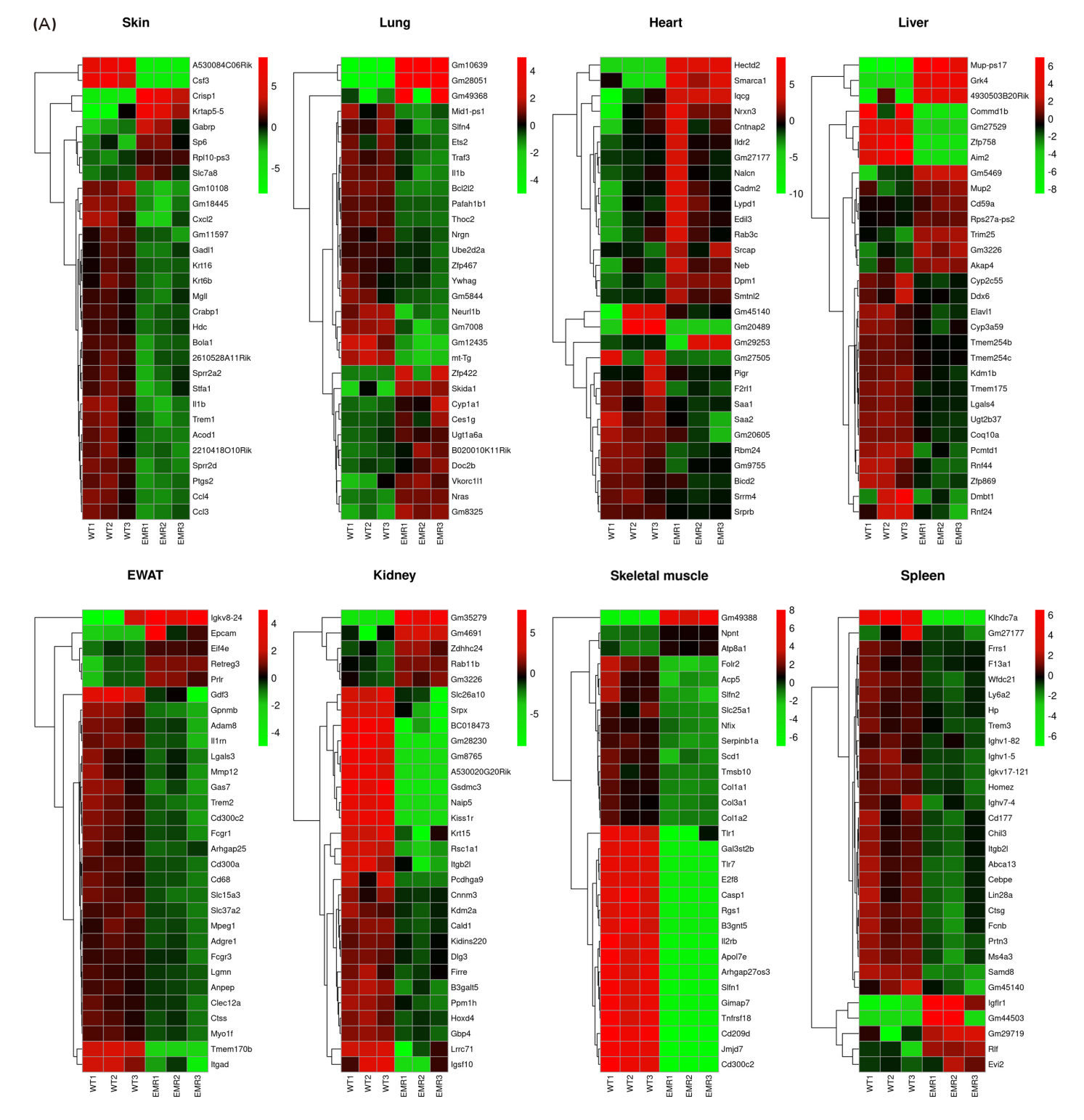

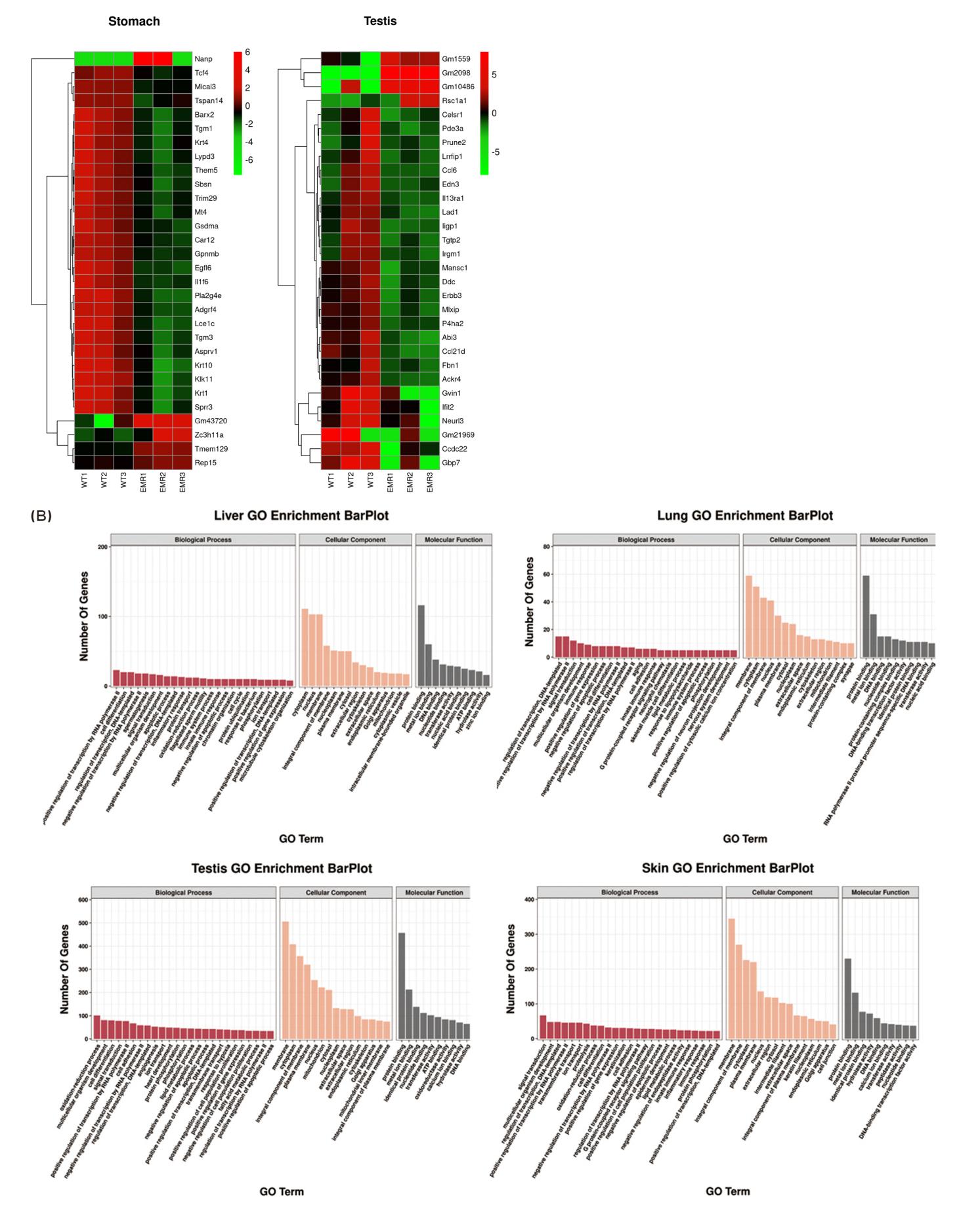

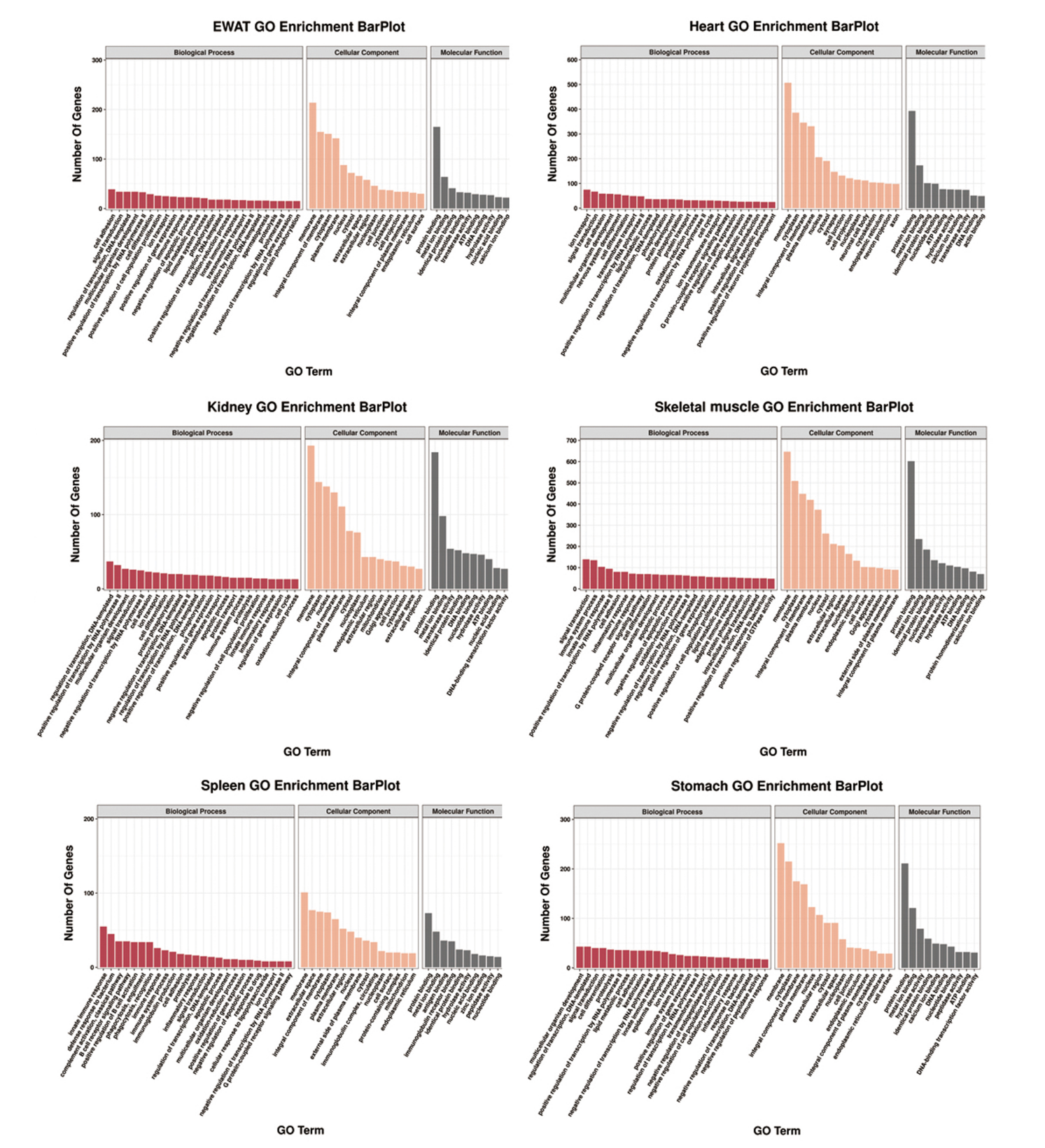

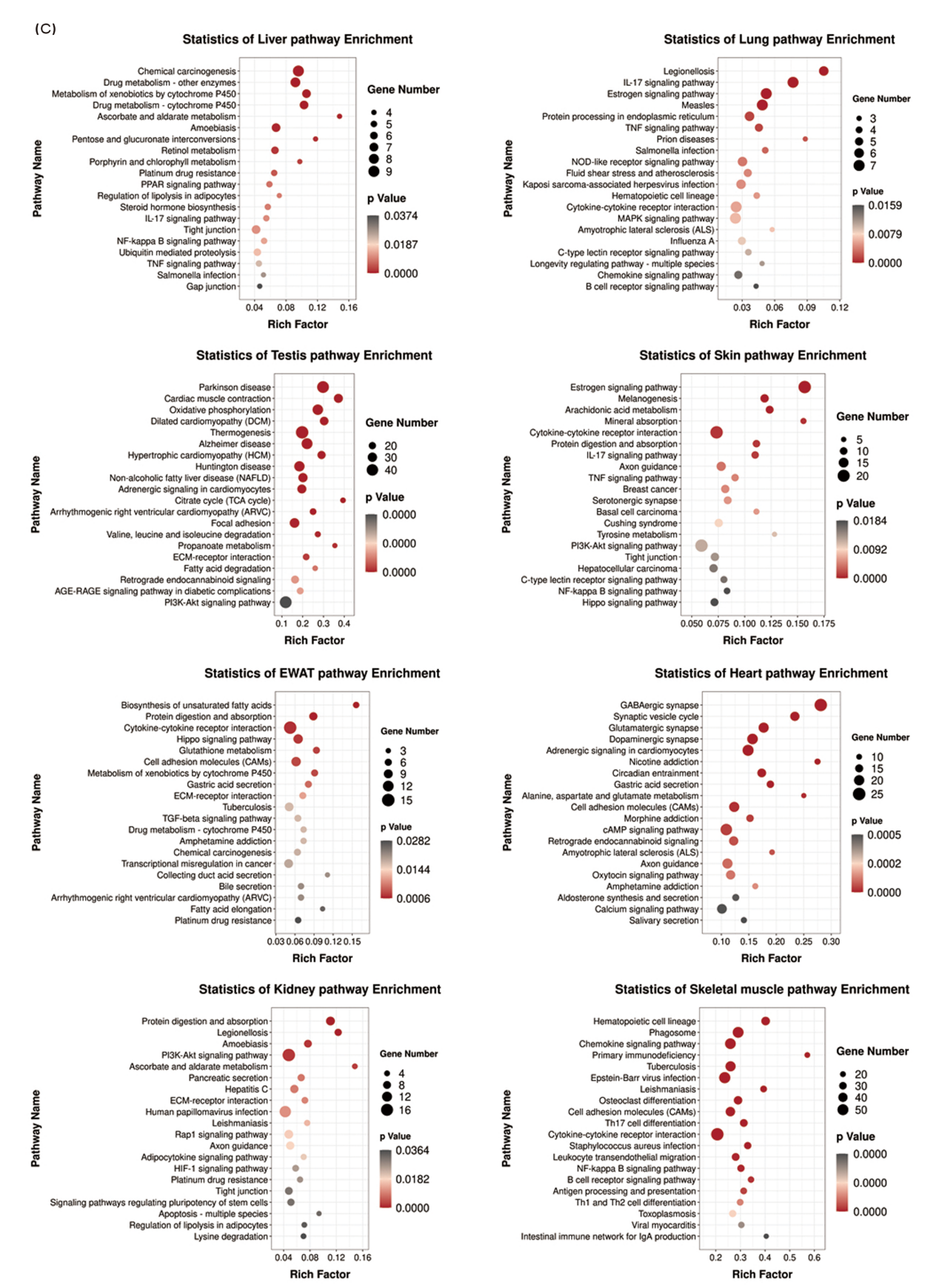

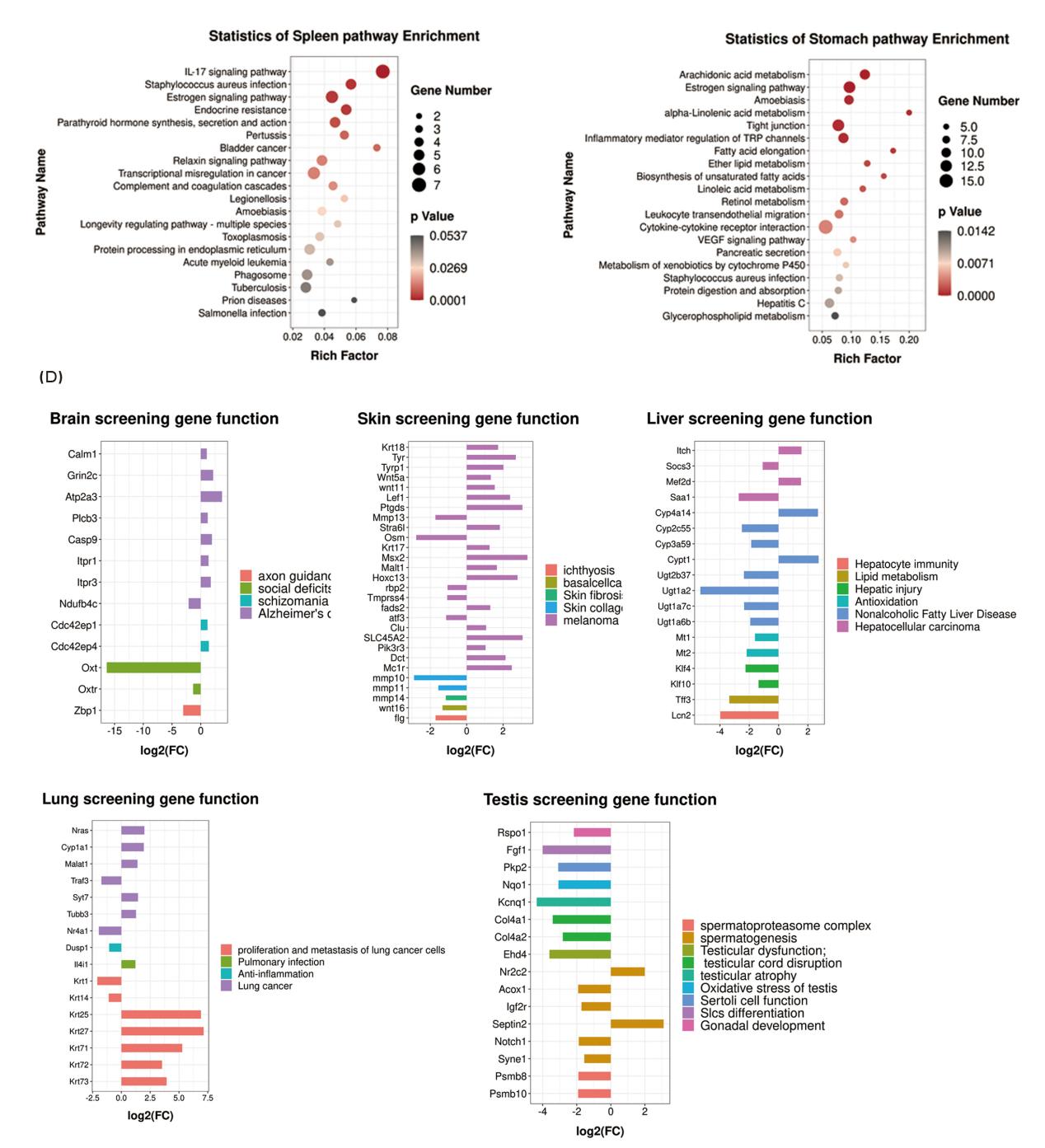


**Figure S3.** Transcriptomic analysis of differentially expressed genes (DEGs) in ten mouse organs. (A) Liver, lung, testis, skin, epididymal white adipose tissue (EWAT), heart, kidney, skeletal muscle (SM), spleen, and stomach heatmaps showing the top 30 (The lowest p-values) up- or down-regulated DEGs (fold change >2 and P < 0.05). (B) Gene Ontology (GO) enrichment of DEGs in 10 organs. (C) Kyoto Encyclopedia of Genes and Genomes (KEGG) enrichment of DEGs in 10 organs. (D) Functional annotations of DEGs in the brain, skin, liver, lung, and testis.


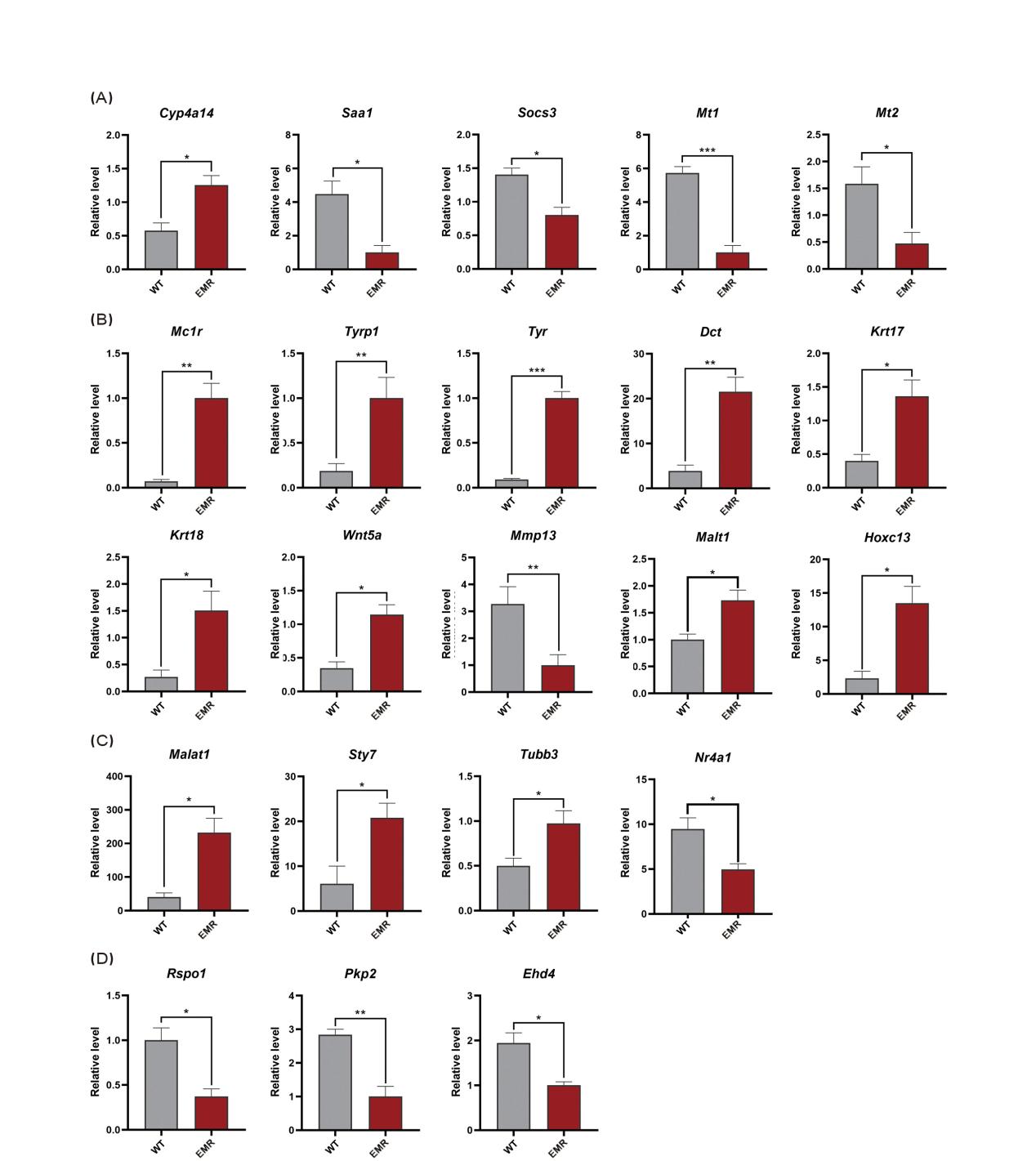


**Figure S4.** Regulatory changes of multiple genes directly related to various diseases in EMR mice. Relative abundances of the genes were determined by reverse transcription quantitative PCR with normalization to *Gapdh* levels (n = 5). Differences were assessed by Student’s *t*-test: **P* < 0.05; ***P* < 0.01; ****P* < 0.001 compared with wild-type (WT) mice.

**
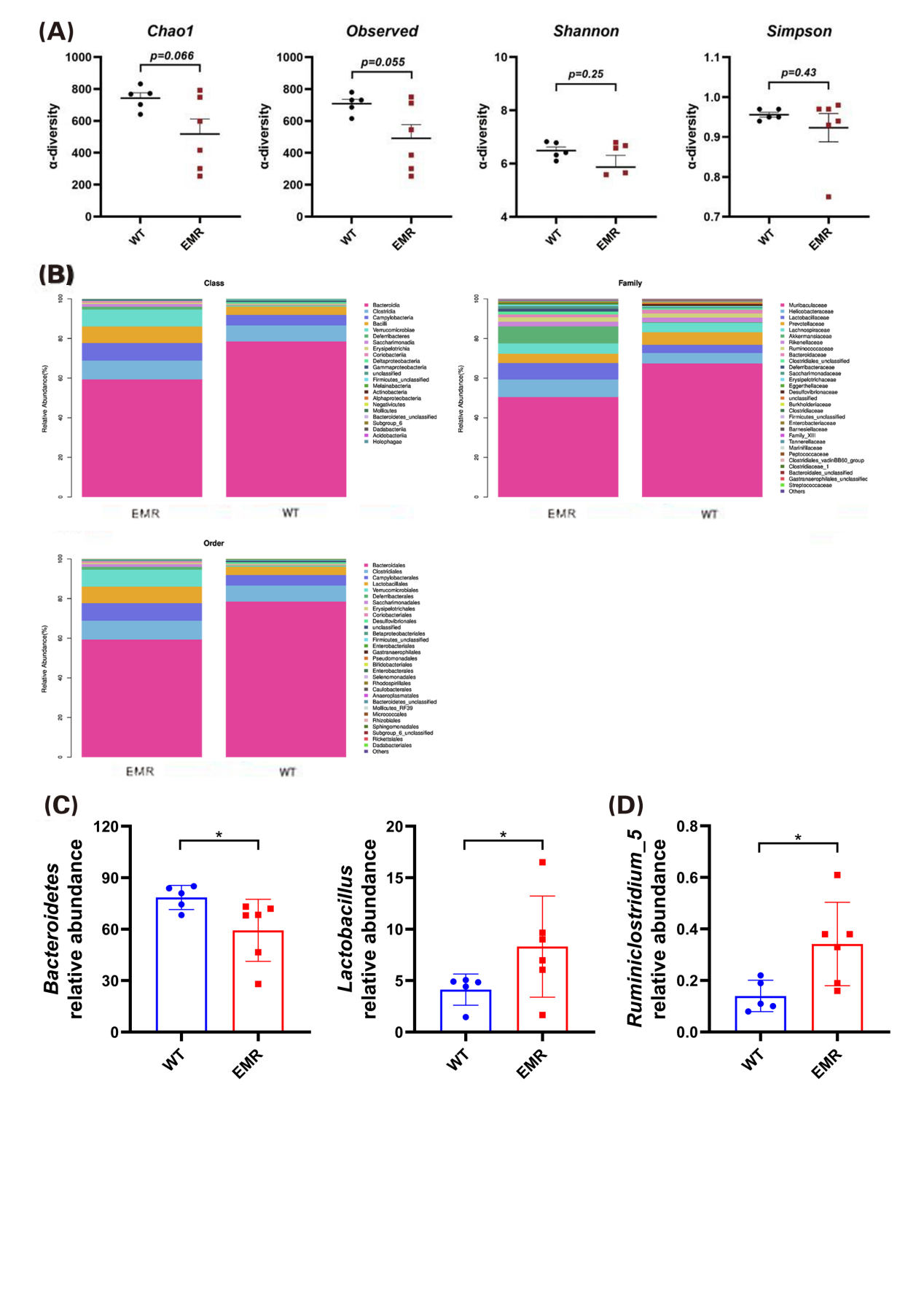
**

**Figure S5.** Alpha diversity and gut microbiota composition in wild-type (WT) and EMR mice. (A) Alpha diversity reflecting species richness and evenness through various indices. (B) Gut microbiota composition at the class, family, and order levels. (C)The relative abundance of *Bacteroidetes* and *Lactobacillus*. (D)The relative abundance of *Ruminiclostridium_5.* **P* < 0.05.


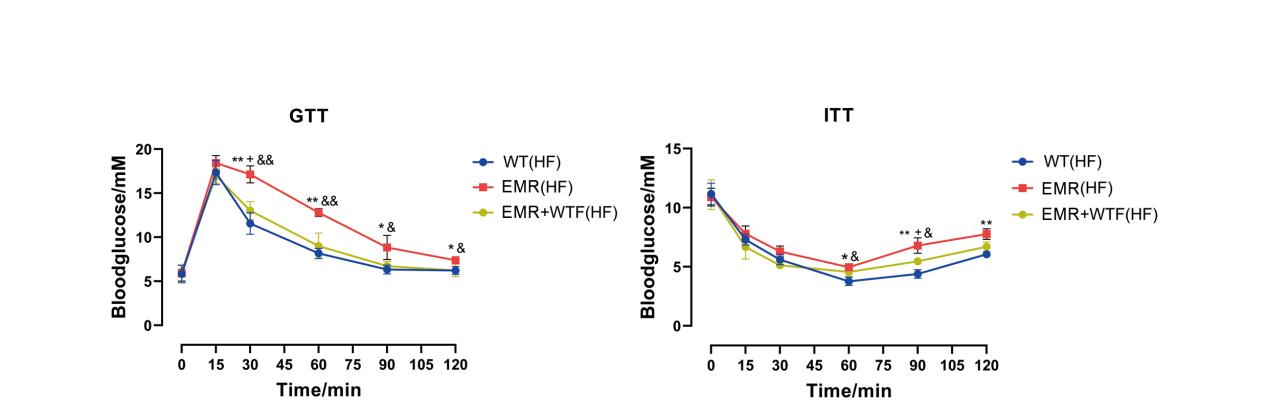


**Figure S6.** Fecal microbiota transplantation (FMT) reversed glucose tolerance and insulin resistance in EMR mice induced by a high-fat diet. Blood glucose concentration was assessed using the GTT and ITT. For comparisons of more than two groups, one-way ANOVA followed by Dunnett’s test was performed. **P* < 0.05, ***P* < 0.01, WT vs EMR; &*P* < 0.05, &&*P* < 0.01, EMR vs EMR+WTF; + *P* < 0.05, WT vs EMR+WTF
